# Spontaneus tonsillar hemorrhage

**DOI:** 10.1016/S1808-8694(15)31082-X

**Published:** 2015-10-22

**Authors:** Savya Cybelle Milhomem Rocha, Alfredo Rafael Dell'Aringa, José Carlos Nardi, Kazue Kobari, Cinthia de Melo

**Affiliations:** 1 Medical 2nd year resident of Otorhinolaryngology at the Marilia Medical College.; 2 Doctor on Otorhinolaryngology, graduated at FMUSP. Head of the Otorhinolaryngology department of the Marilia Medical College.; 3 Master's degree on Otorhinolaryngology, graduated at FMUSP-RP. Teacher of Otorhinolaryngology at the Marilia Medical College.; 4 ENT specialist, Teahcer of Otorhinolaryngology at the Marilia Medical College.; 5 Medical 3rd year resident of Otorhinolaryngology at the Marilia Medical College. Marilia Medical College - FAMEMA.

**Keywords:** peritonsillar abscesses, von willebrand's disease, spontaneous tonsillar hemorrhage, infectious mononucleosis, measles

## INTRODUCTION

Spontaneous tonsillar hemorrhage is a rare event; most of these cases have been the result of infectious tonsillitis.1 There are reported cases of spontaneous tonsillar hemorrhage in medical literature associated with bacterial infection, measles virus infection, infectious mononucleosis, peritonsillar, parapharyngeal and retropharyngeal abscesses and, less frequently, vascular malformation, aneurisms or pseudoaneurisms of the carotid and superficial temporal arteries, von Willebrand's disease and local or regional cancer.^1^,^2^

In a review of literature, Lourenço et al.^1^ found 21 cases of spontaneous tonsillar hemorrhage resulting from acute tonsillitis. Cases of spontaneous hemorrhage have been reported in peritonsillar abscesses, mostly when spontaneous drainage occurred during the pre-antibiotic era.^1^

The prevalence of hemorrhage associated with infectious mononucleosis is 3 to 6.9%; of these, 2.2% presented oropharyngeal hemorrhage.^1^,^2^,^3^ Thrombocytopenia is associated with this condition, but hemorrhage may result only due to local inflammation, necrosis and erosion of superficial tonsillar blood vessels.^1^

In measles, hemorrhagic complications are uncommon. However, there is a rare variant known as hemorrhagic measles that affects mostly immunocompromised patients.^4^

Tonsillar hemorrhage is a rare finding in von Willebrand's disease. There are 2 cases reported in literature where tonsillar hemorrhage was the first manifestation of this disease.^5^

An explanation of the pathophysiology of this type of hemorrhage is that acute inflammation results in increased blood flow to the tonsils, secondary edema, and vascular congestion; during the local inflammatory process, superficial dilated blood vessels undergo necrosis and bleed.^1^

In the pre-antibiotic era these hemorrhages were fatal; they were commonly due to erosion of major vessels, secondary do deep abscesses. Currently, most tonsillar hemorrhages are mild and result from the bleeding of superficial peripheral blood vessels.^1^

We report the following case to discuss the etiology and clinical strategies in spontaneous tonsillar hemorrhage.

## CASE REPORT

A female patient aged 13 years was taken to the emergency unit of the FAMEMA Child Emergency Hospital (Pronto-Socorro Infantil da FAMEMA) with oral hemorrhage lasting 15 hours. She denied odynophagia, fever, local trauma, or coughing with sputum. She reported having been treated for bacterial tonsillitis with endovenous ampicillin one month ago. She reported a history of repeat otitis and tonsillitis during childhood. There were no reports of other hemorrhages in her clinical history or that of her family.

The otorhinolaryngological exam revealed tonsillar hypertrophy (+++/++++) according to Brodsky's scale, and bleeding from the superior pole of the right tonsil. Laboratory work-up showed normal coagulation tests, leukocytosis (16.400/mm^3^) with predominant segmented cells (92%) on the complete blood count, and negative serology for syphilis and infectious mononucleosis.

Antibacterial treatment was started using cephalothin and metronidazole associated with a diet containing cold food to control local bleeding. Follow-up has been uneventful, with no further episodes of tonsillitis so far. Exams to investigate von Willebrand's disease were requested, but the patient has not returned to the hospital.

## DISCUSSION

Most of these patients are aged between 20 and 30 years.3 There is no gender predominance.1 The average duration of symptoms is 2 to 5 days.3 The case above showed sudden bleeding which had lasted one day. The superior tonsillar pole is the most frequently involved site for hemorrhage,1,3 as in this case.

On being examined, half of these patients present active bleeding or blood clots. Tonsillar hemorrhage frequently involves a single tonsil.^1^

According to Rios et al.,3 recurring tonsillitis is the main predisposing factor leading to spontaneous tonsillar hemorrhage, with infection as the main etiology. The patient above had a history of recurring tonsillitis.^1^

Currently most of the cases of spontaneous tonsillar hemorrhage are associated with peripheral blood vessel erosion, which is the case of our patient. ^1^

Treatment includes local control of bleeding with chemical cauterization, electrocoagulation or nebulizing with adrenalin. Tonsillectomy during active bleeding is rarely indicated except for cases of recurring tonsillitis.^1^,^6^

## CONCLUSION

Acute bacterial tonsillitis is currently the most important cause of spontaneous tonsillar hemorrhage. The incidence is 1.1%.^3^

The etiology of bleeding should be investigated. Exams such as a complete blood count and coagulation tests are not the ideal exams for a final diagnosis, but may be useful.

Most authors recommend local control of bleeding; tonsillectomy is rarely indicated.
